# Whole-genome sequencing of single circulating tumor cells from neuroendocrine neoplasms

**DOI:** 10.1530/ERC-21-0179

**Published:** 2021-07-16

**Authors:** Alexa Childs, Christopher D Steele, Clare Vesely, Francesca M Rizzo, Leah Ensell, Helen Lowe, Pawan Dhami, Heli Vaikkinen, Tu Vinh Luong, Lucia Conde, Javier Herrero, Martyn Caplin, Christos Toumpanakis, Christina Thirlwell, John A Hartley, Nischalan Pillay, Tim Meyer

**Affiliations:** 1 UCL Cancer Institute, University College London, London, UK; 2 Department of Histopathology, Royal Free London NHS Foundation Trust, London, UK; 3 Department of Gastroenterology, Royal Free London NHS Foundation Trust, London, UK; 4 Department of Oncology, Royal Free London NHS Foundation Trust, London, UK; 5 Research Department of Pathology, Cancer Institute, University College London, London, UK; 6 Department of Cellular and Molecular Pathology, Royal National Orthopaedic Hospital NHS Trust, Stanmore, Middlesex, UK

**Keywords:** neuroendocrine tumors, circulating tumor cells, single cell, copy number variation, whole-genome sequencing

## Abstract

Single-cell profiling of circulating tumor cells (CTCs) as part of a minimally invasive liquid biopsy presents an opportunity to characterize and monitor tumor heterogeneity and evolution in individual patients. In this study, we aimed to compare single-cell copy number variation (CNV) data with tissue and define the degree of intra- and inter-patient genomic heterogeneity. We performed next-generation sequencing (NGS) whole-genome CNV analysis of 125 single CTCs derived from seven patients with neuroendocrine neoplasms (NEN) alongside matched white blood cells (WBC), formalin-fixed paraffin-embedded (FFPE), and fresh frozen (FF) samples. CTC CNV profiling demonstrated recurrent chromosomal alterations in previously reported NEN copy number hotspots, including the prognostically relevant loss of chromosome 18. Unsupervised hierarchical clustering revealed CTCs with distinct clonal lineages as well as significant intra- and inter-patient genomic heterogeneity, including subclonal alterations not detectable by bulk analysis and previously unreported in NEN. Notably, we also demonstrated the presence of genomically distinct CTCs according to the enrichment strategy utilized (EpCAM-dependent vs size-based). This work has significant implications for the identification of therapeutic targets, tracking of evolutionary change, and the implementation of CTC-biomarkers in cancer.

## Background

The molecular characterization of tumors has advanced our understanding of the major somatic driver mutations and informed the development of targeted therapies, which have transformed outcomes in selected patient populations (Vogel *et al.* 2002, Sharma *et al.* 2007, Sosman *et al.* 2012). Whilst tissue biopsy remains central to diagnostic work-up, it is invasive, limited by the overall percentage of tumor cells, and subject to heterogeneity exhibited in primary and metastatic tumors (Navin *et al.* 2010, Gerlinger *et al.* 2012, Walter *et al.* 2018). Furthermore, bulk genomic analysis cannot provide resolution at the single-cell level, which is required to fully define the extent of tumor heterogeneity.

Technological advances in whole-genome amplification (WGA) and next-generation sequencing (NGS) methods now permit genomic analysis at the single-cell level and are uniquely placed to unravel complex clonal structures and track tumor evolution over time. Furthermore, characterization of single-circulating tumor cells (CTCs) as part of a minimally invasive 'liquid biopsy' provides an opportunity to explore tumor biology and identify therapeutic targets.

The first clinical applications of CTCs focused on enumeration using the EpCAM-dependent CellSearch® platform, which has been shown to be both prognostic and predictive across a wide range of epithelial malignancies (Cristofanilli *et al.* 2004, Cohen *et al.* 2008, de Bono *et al.* 2008, Krebs *et al.* 2011, Poveda *et al.* 2011), including neuroendocrine neoplasms (Khan *et al.* 2011, 2013, 2016, Mandair *et al.* 2021). More recently, molecular analysis of single CTCs has been used to identify predictive biomarkers, such as the T790M resistance allele in NSCLC (Maheswaran *et al.* 2008). In SCLC, a pretreatment CTC-based biomarker has been shown to predict sensitivity to first-line chemotherapy (Carter *et al.* 2017).

Neuroendocrine neoplasms (NEN) represent a heterogeneous disease entity with diverse histology, clinical features, and prognosis (Dasari *et al.* 2017). They are characterized by a low mutational burden (Banck *et al.* 2013), but recurrent patterns of copy number variation (CNV) have been observed (Kulke *et al.* 2008, Cunningham *et al.* 2011, Capurso *et al.* 2012). CNVs affect a greater portion of the cancer genome than any other somatic genetic alteration (Heitzer *et al.* 2016), and CNV burden is prognostic for cancer-free and overall survival in multiple tumor types (Hieronymus *et al.* 2018) including NEN, where aneuploidy can be used to define distinct molecular subgroups of prognostic relevance (Karpathakis *et al.* 2016).

In this study, we perform CNV analysis of single NEN CTCs, aiming to define the extent of genomic heterogeneity both within and between patients and to compare single-cell CTC data with bulk tissue analysis. CTC enrichment in NEN patients has to date been confined to EpCAM-dependent methodologies, which may fail to capture the full diversity of CTCs seen in this disease (Gorges *et al.* 2012). Here, we utilize both the EpCAM-based CellSearch and epitope-independent Parsortix® systems in order to interrogate the full diversity of cells at the CNV level and investigate whether single-cell CTCs may differ at a genomic level, according to EpCAM expression.

## Methods

### Patients

NEN patients were recruited at the Royal Free Hospital, London, between September 2014 and February 2018. The study was approved by the Local Ethics Committee (NRES Committee London – Bromley, IRAS ref 13/LO/0376), and all participants were required to provide written informed consent. Eligible patients had a histologically confirmed diagnosis of metastatic NEN in the absence of any other active malignancy. Tumors were graded according to the European Neuroendocrine Tumor Society (ENETS) guidelines (Bosman *et al.* 2010).

### CTC enrichment using CellSearch

Peripheral blood samples (7.5 mL) were collected into CellSave tubes (Veridex LLC), stored at room temperature, and processed within 96 h using the Celltracks Autoprep and Analyzer II platform for the semi-automated staining, enrichment, and the enumeration of CTCs as previously described (Cristofanilli *et al.* 2005, Riethdorf *et al.* 2007). CTCs were defined as cells with a DAPI positive nucleus and positive EpCAM and cytokeratin expression in the absence of CD45 staining. All evaluations regarding enumeration of CTCs were made by two independent operators without the knowledge of patient pathology. Enriched samples were re-suspended, aspirated from the CellSearch cartridge, and stored at −20°C in 50% glycerol.

### CTC enrichment using Parsortix

Blood was collected in Streck tubes (10 mL) and incubated for 24–48 h prior to size-based enrichment with the Parsortix platform (ANGLE) using software protocols provided by the manufacturer. Following enrichment, samples were harvested in a total volume of 1.2 mL of HBS by applying a reverse flow to the separation cassette. Enriched samples were re-suspended in 200 μL of autoMACS running buffer and fixed and stained for further processing on a sterile transwell polycarbonate membrane insert placed within a 50mL Falcon tube. BSA of 3% (200 μL) was pipetted to entirely cover its surface for a 10 min incubation. The 50 mL tube was centrifuged at 500 g for 2 min to elute the BSA solution from the filter prior to transferring the enriched patient sample onto the insert surface. One hundred microliter of a 10% CD45 staining solution (10 μL anti-CD45-APC (Miltenyi Biotec) and 90 μL of running buffer) and 100 μL of a 10% CK staining solution (10 μL anti-CK-PE (Abcam) and 90 μL Inside Perm (Miltenyi Biotec)) were used to sequentially stain samples for CD45 and cytokeratin prior to staining for nuclear content using 100 μL of a 0.001 mg/mL solution of Hoechst 33342 (Sigma–Aldrich). After washing with SB115 buffer, the cell suspension was transferred into a sterile 1.5 mL tube prior to volume reduction and loading into the DEPArray™ cartridge.

### Cell isolation from FFPE

FFPE tissue sections of 40–60 μm thickness were dissociated into single-cell suspensions and stained as previously described (Bolognesi *et al.* 2016). To enable visualization and identification of cells using the DEPArray, cytokeratin and vimentin were used as tumor and stromal cell markers, respectively. Cell suspensions were stained with anti-cytokeratin MNF116 (IgG1) (DAKO), anti-cytokeratin AE1/AE3 (IgG1) (Millipore–Chemicon), and anti-Vimentin 3B4 (IgG2A) (DAKO).

Dissociated FFPE samples were subjected to a DNA quality-control assay using the DEPArray FFPE QC kit (Silicon Biosystems). Each sample was given a QC score between 0 and 1 based on a qPCR-based assay. Samples with a sufficiently high DNA quality as determined by a QC score ≥ of 0.4 according to manufacturer’s guidelines were processed on the DEPArray platform for retrieval of single tumor cells.

### Cell isolation from fresh tissue

Fresh tissue samples were collected in RPMI 1640 medium (Gibco) and processed within 3 h of collection. The tumor sample was placed in 1mL of dissociation solution (240 μL collagenases, 150 μL DNAse, and 13.85 mL of RPMI media) and processed in a gentleMacs dissociator for one cycle, followed by two consecutive 30 min incubations at 37°C. Single-cell suspensions were created using a 50 μL cell strainer and centrifuged and re-suspended in 5mL of RPMI prior to re-suspending in 1mL of freezing medium (10% DMSO in FBS) for storage at −80°C. Samples were fixed with 2% paraformaldehyde (Fluka) for 20 min at room temperature prior to staining for cytokeratin, vimentin, and DAPI performed as per FFPE samples.

### DEPArray sorting and recovery

Both CellSearch- and Parsortix-enriched samples were imaged and sorted using the DEPArray system (Silicon Biosystems) as per the manufacturer’s instructions (Abonnenc *et al.* 2013). Image-based selection was used to identify and recover individual cells of interest as either single cells or pools of cells, based on their morphological features, DNA content, and fluorescence labeling; CTCs (CK-PE^+^/CD45-APC^−^/DAPI^+^) and white blood cells (WBC) (CK-PE^−^/CD45-APC^+^/DAPI^+^).

For analysis of FFPE samples with the DEPArray, between 5000 and 10000 stained cells were loaded into the cartridge, and cell sorting was executed according to DEPArray User’s Manual rev 1.1_sw 2.1.1. The cytokeratin+ vimentin- tumor cell population and cytokeratin-vimentin+ stromal cell population were gated separately to evaluate morphology and staining characteristics prior to selecting cells for recovery.

### Whole-genome amplification of single-cell DNA and quality-control assay

WGA was performed on all recovered single-cells using the Ampli1™ WGA kit version 02 (Silicon Biosystems) as per the manufacturer’s instructions to generate a 50 μL WGA product. For single cells derived from blood (CTCs and WBC) and fresh tissue (tumor and stromal cells), the quality of the WGA product was determined using the Ampli1 QC Kit (Silicon Biosystems). A genomic integrity index (GII) was allocated for each sample, scored from 0 to 4. Only single cells with sufficiently good quality DNA as determined by a GII ≥ 2 were selected for downstream analysis.

### Nucleic acid extraction

For bulk sequencing, DNA was extracted from 5 to 10 sections of 10 μm thickness from three FFPE blocks using the DNAstorm FFPE DNA Isolation Kit (CELLDATA) following the manufacturer's instructions. DNA was eluted into 75 μL of nuclease-free water and concentrations were measured using the NanoDrop-1000 Spectrophotometer (NanoDrop) and Qubit 2.0 Fluorometer (Invitrogen). Hematoxylin and eosin-stained sections were evaluated to ensure >80% purity of tumor specimens prior to processing.

### Lowpass whole-genome sequencing and bioinformatics

Ampli1 LowPass kit for Illumina (Menarini Silicon Biosystems) was used for preparing low-pass whole-genome sequencing (WGS) libraries from single cells. For high-throughput processing, the manufacturer’s procedure was implemented in a fully automated workflow on a STARlet Liquid Handling Robot (Hamilton). Ampli1 LowPass libraries were normalized and sequenced by HiSeq 2500 instrument using 150 SR rapid-run mode. The obtained FASTQ files were aligned to the hg19 human reference sequence using Burrows–Wheeler Aligner version 0.7.12 (BWA). Copy number alterations in the data were identified using Control-FREEC software (version 11.0).

For bulk analysis of FFPE samples, genomic DNA was quantified using Qubit 3 fluorometer with dsDNA BR kit according to the manufacturer’s instructions. One microgram of genomic DNA was used to prepare whole-genome sequencing libraries using Nonacus Cell 3 Target: Library Preparation kit. Library preparation was done according to the manufacturer’s instructions. Enzymatic fragmentation was performed at 32°C for 14 min to obtain library fragments with an average size of 250 bp followed by ligation of UMI Adapters on both ends of the 5’-phosphorylated/3’-dA-tailed DNA fragments. Libraries were purified using Target Pure NGS clean-up beads, and minimal PCR amplification was carried out using four cycles of amplification. Libraries were quantified using Qubit 3 fluorometer with dsDNA BR kit and run on an Agilent Bioanalyzer DNA 1000 chip according to the manufacturer’s instructions. Average library fragment length was determined from the bioanalyzer trace. Library molar concentration was determined based on the average fragment size and the Qubit concentration. All libraries were normalized to 10 nM working concentration and pooled. The dual-indexed library pool was sequenced on Illumina Nextseq 500/550 platform to generate paired-end reads. The Nonacus Cell 3 Target: Library preparation protocol adds unique molecular identifiers (UMIs) to the sequencing libraries which were sequenced by additional nine cycles of sequencing added on to the i7 index read.

Bulk sequencing data were processed with the nextflow Sarek v2.3.FIX1 pipeline (https://github.com/UCL-BLIC/Sarek_v2.3.FIX1) following GATK best practices. Specifically, reads were aligned against hg38 with BWA v0.7.17, duplicated reads were marked, and reads were recalibrated with GATK v4.1.1.0. CNV profiles were obtained by running Control-FREEC v11.5 with WGS recommended parameters.

### Statistics

All statistical analyses were performed in R. Pairwise Manhattan distances were calculated for all samples, using only copy number bins that were not NA for each pair. Hierarchical clustering of copy number profiles using these distances was performed with Ward’s minimum variance method.

When comparing bulk and CTC copy number profiles, the mean copy number across CTC copy number bins that overlapped a bulk bin was taken. Any bulk bin without an overlapping CTC bin was not given a copy number designation.

*t*-Distributed stochastic neighbor embedding (TSNE) analysis was performed using the R package Rtsne, using only the genomic bins that were non-missing for all samples analyzed, with a perplexity of 30.

Correlations between copy number profiles were calculated with respect to a base copy number of 2, as described in Gao *et al.* (2017):


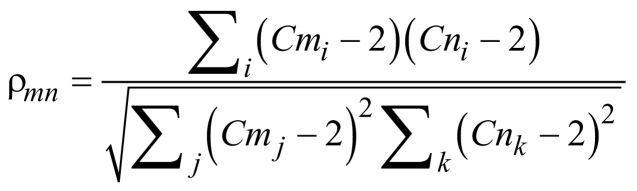


where ρ*_mn_* is the correlation between samples *m* and *n*, while *Cm_i_* is the copy number for sample *m* at bin *i*.

To account for differences in ploidy, correlation was also calculated with respect to the average copy number across all bins for each sample:


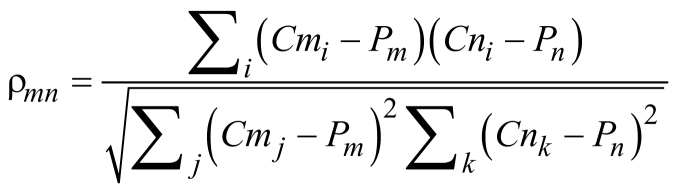


where *P_m_* is the mean copy number for sample *m* across all bins.

Metrics chosen to investigate copy number dynamics within a sample were the proportion of genome altered (number of CN! = 2 bins divided by the total number of bins) and Shannon’s diversity index, 
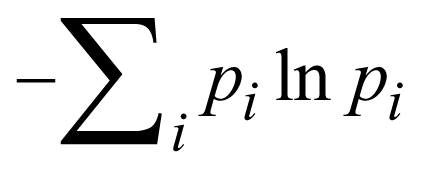
, where *p_i_* is the proportion of copy number bins with copy number state *i,* that is, CN = 2. Tests for statistical differences between distributions for these metrics were performed using the Kolgomorov–Smirnov test.

Copy number gains and losses were defined in relation to ploidy. Gains were defined as log_2_(CN/ploidy) > 0.9, while losses were defined as log_2_(CN/ploidy) < −0.9. The proportion of cells with a loss at a given genomic bin was used as a metric for a single patient. When combining multiple patients, the mean proportion of cells across all patients considered was used. A threshold for statistically significant recurrent gain or loss was determined by bootstrapping the original copy number data; for each patient, copy number states were sampled with replacement from every copy number state seen in the original data for that patient, this was performed for the same number of cells as were originally profiled for that patient. Gains and losses were defined as previously, and the proportion of simulated cells with a gain/loss at each genomic bin was calculated. This was repeated 1000 times per patient, and the threshold for determining recurrent gains/losses was set as 99.9th percentile value across all genomic bins for gains or losses separately. For a threshold where multiple patients are being considered, the same bootstrapping was performed for each patient, but the threshold was determined as the 99.9th percentile of the mean proportion of cells with gain/loss across the patients being evaluated.

## Results

### Patient characteristics and sample collection

**Figure 1:**
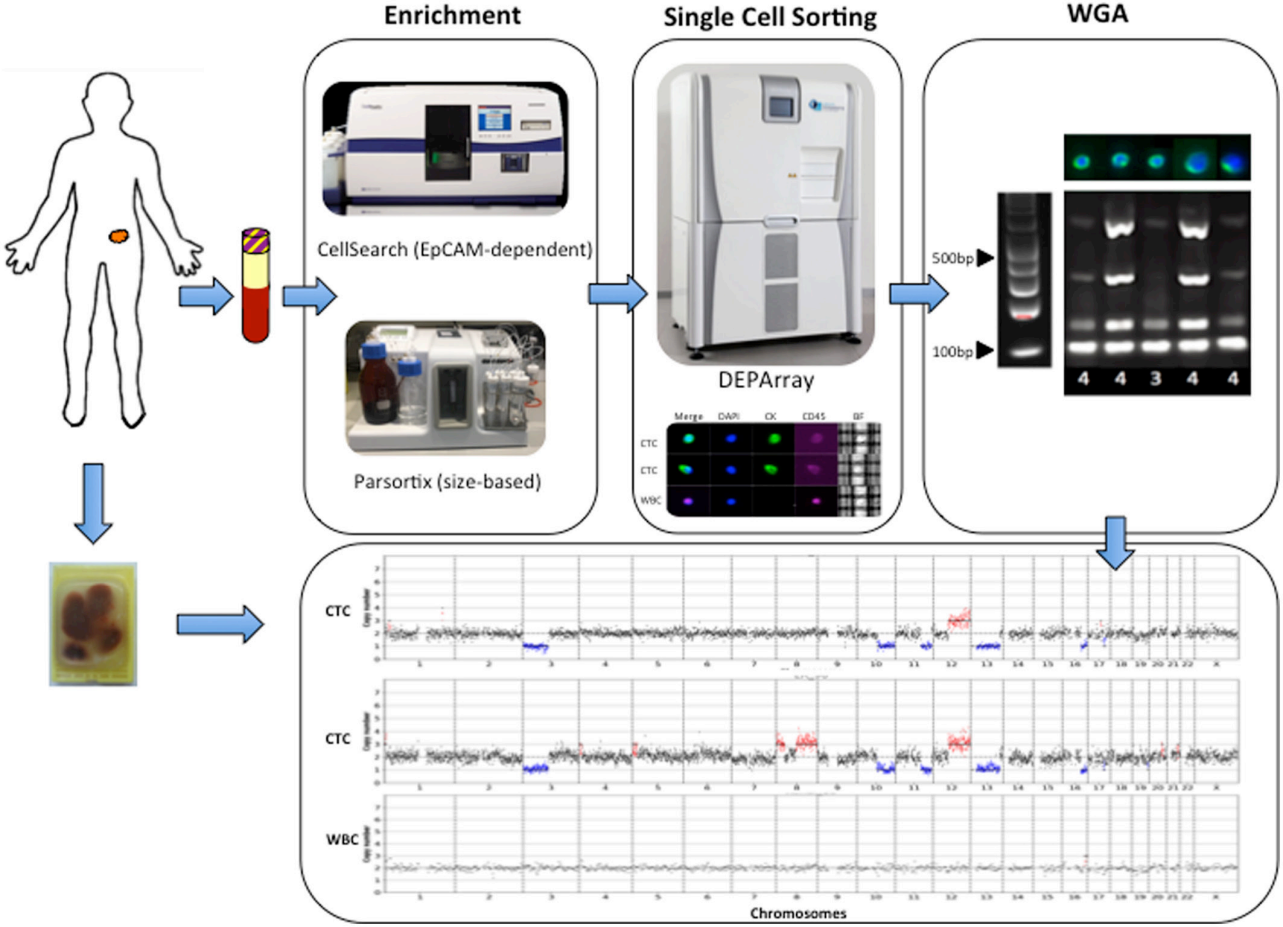
Experimental design of the study. Workflow used in the study to enrich for CTCs and CNV profiling using Ampli1 WGA and LowPass kit for Illumina. Following enrichment (EpCAM-dependent vs size-based platforms), single NEN CTCs and matched WBC are selectively recovered in dynamically controlled dielectrophoretic cages using the DEPArray Image-Assisted Digital Cell Sorter. CTC samples undergo WGA and QC prior to low-resolution whole-genome sequencing for CNV profiling. Where surgical resection or biopsy specimens are available, samples are processed for bulk LPWGS and single-cell LPWGS as per CTCs.

Seven NEN patients were included with primary tumor sites comprising the small intestine (SINET) (*n* = 4), pancreas (*n* = 1), gastro-esophageal junction (GOJ) (*n* = 1), and kidney (*n* = 1). All patients had peripheral blood samples taken for CTC enrichment using the EpCAM-dependent CellSearch platform, and three patients had concomitant samples enriched using the size-based Parsortix device (Fig. 1). Blood samples were taken from new patients at the time of the first presentation to our clinic (patients 1, 3, 5, 6) or at the time of disease progression prior to commencing systemic therapy (patients 2, 4, 7). Matched WBC were analyzed as negative controls. A total of seven tissue samples (6 FFPE, 1 FF) from six patients were analyzed. Of the seven samples, four were primary tumor samples (3 small intestine, 1 GOJ) and three were metastatic sites (2 liver, 1 brain). One patient (patient 1) had no available tissue for analysis. The clinical and treatment characteristics as well as the samples analyzed per patient are summarized in Table 1.

**Table 1:** Summary of clinical characteristics.

Patient ID	Sex	Age	Primary site	Grade	Treatment	CellSearch CTCs	Parsortix CTCs	WBC	Fresh tissue single cells	FFPE single cells	FFPE bulk samples
1	M	74	Small intestine	2	na	4	na	4	na	na	na
2	F	45	Small intestine	3	PEN-221	15	5	na	na	2	Pituitary metastasis
3	F	69	Small intestine	2	na	7	8	na	8	na	Small bowel
4	M	65	Small intestine	1	SSA	18	na	na	na	na	Small bowel
5	M	47	Pancreas	2	na	12	na	4	na	na	na
6	M	64	GOJ	3	na	11	na	na	na	1	na
7	F	33	Renal	2	PEN-221	21	11	10	na	2	na

All tissue samples are FFPE unless specifically indicated otherwise.

F, female; FFPE, formalin-fixed paraffin-embedded; GOJ, gastro-esophageal junction; M, male; na, not applicable; SSA, somatostatin analogs; PEN-221, novel antibody-drug conjugate.

### CTC sequencing

**Figure 2:**
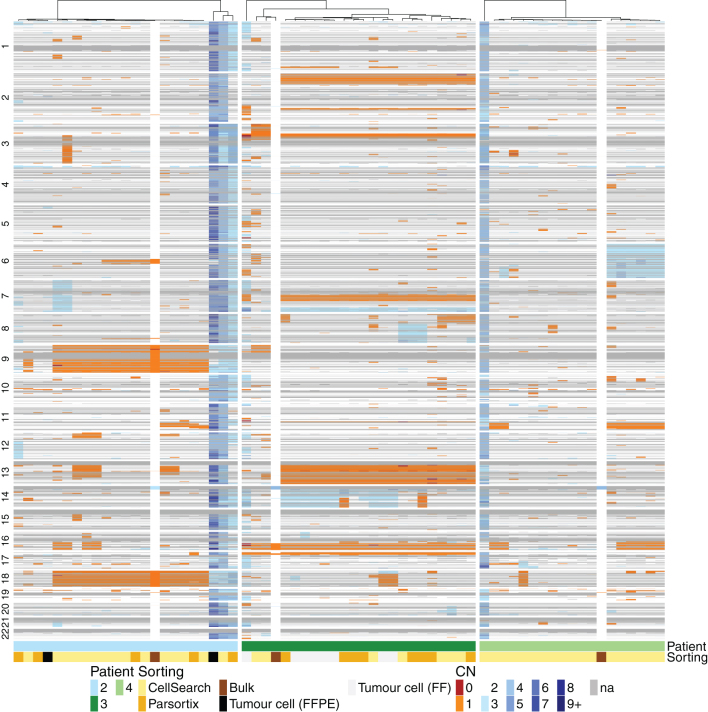
Comparison of low-resolution whole-genome copy number profiles for CTCs and bulk tissue reveals reproduction of the majority of the CNV from the formalin-fixed paraffin-embedded (FFPE) and fresh frozen (FF) tissue in CTC samples. Unsupervised hierarchical clustering heat map of each analyzed individual CTC and tissue sample based on CNV from three SINET patients. Each patient is depicted with one color as shown on the phenobar at the bottom of the heat map. Individual CTCs are categorized according to enrichment method and tissue into bulk vs single-cell FFPE (see key). Chromosomal CNV is shown from top to bottom for each individual cell or sample; copy number gains are depicted in blue, losses in orange.

**Figure 3:**
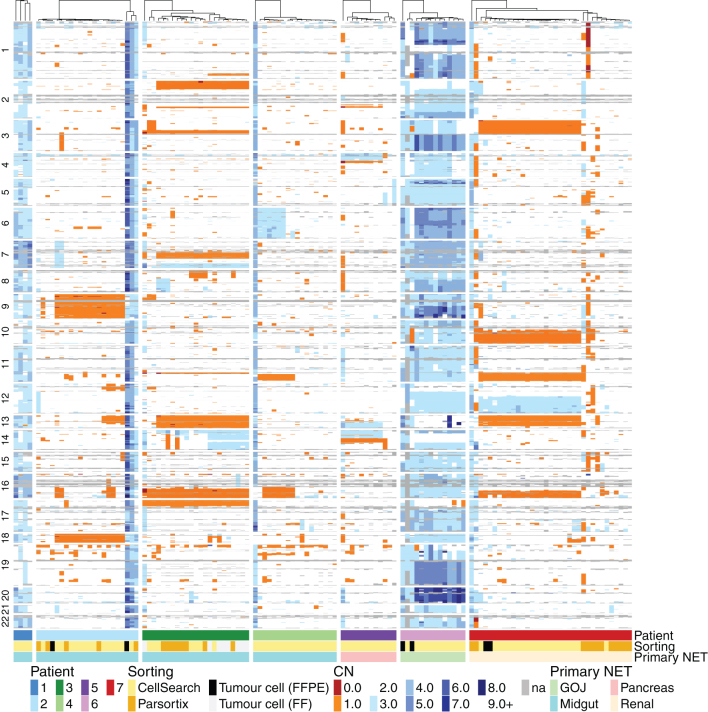
Individual CTC CNV data depicting complex intrapatient and interpatient genomic diversity. Unsupervised hierarchical clustering heat map of all analyzed CTCs based on CNV across seven patients. Each patient is depicted with one color as shown on the phenobar at the bottom of the heat map along with the cell sorting method and primary NET site.

In total, 125 single CTCs were isolated from seven patients and successfully subjected to the whole-genome amplification (WGA), quality-control PCR, and low-pass whole-genome sequencing (LPWGS). Single CTCs displayed high-quality metrics, with only 3.5% failing to pass the quality checks for single-cell CNV. As a control, 17 single WBC (CD45 positive cells) were isolated and subjected to the same procedures. CD45 positive cells showed balanced copy number profiles (Supplementary Fig. 1, see section on supplementary materials given at the end of this article) whereas CTCs showed multiple gains and losses (Figs 2 and 3), confirming the aberrant nature of these tumor cells and the uniformity of single-cell WGA with the Ampli1 kit. The sensitivity and specificity of CTC identification and recovery by the DEPArray were assessed across all single cells subjected to LPWGS. Cells with CNV profiles demonstrating an overabundance of substantial chromosomal gains and losses were considered CTC, whilst cells demonstrating flat profiles were classified as WBC (Ferrarini *et al.* 2018, Mangano *et al.* 2019). Using CNV profiles as the ultimate classifier of cell status, DEPArray selection had a positive predictive value of 95% and a negative predictive value of 100% (*P* < 0.0001).

Single tumor cells derived from FFPE surgical specimens/biopsies were also subjected to the same procedures as CTCs. DNA quality of single-cell suspensions was assessed using the Ampli1 QC Kit (Silicon Biosystems) prior to cell sorting. Four of the seven samples had QC values ≥ 0.4 indicating a sufficient DNA quality for single-cell CNV analysis, and eight to ten single cells from each sample were processed for CNV analysis. The majority of single tumor cells had high derivative log ratio spread values in keeping with low library quality and only 15% of recovered single cells yielded sufficient quality results for CNV analysis.

### CTC vs tumor tissue CNV profiles

For the three patients with sufficient matched FFPE tissue available for bulk analysis, whole-genome CNV profiles were compared between CTCs and bulk FFPE samples (Fig. 2). The CNVs demonstrated in bulk tissue analysis were predominantly losses and these were also detectable in most CTCs. For example, in patient 2, losses in chromosomes 6, 9, and 18 are seen in bulk tissue and in 25, 80, and 65% of CTCs respectively, while patient 3, losses in chromosome 16 were observed in bulk tissue and 100% of CTCs (Fig. 2). The majority of these concordant genomic losses are located in regions of the genome previously described as altered in NENs, with loss of chromosomes 9 and 18 reported in 20% and 60–78% of SINETS, respectively. However, single CTC data demonstrated the presence of clones enriched in additional somatic copy number alterations not detectable at the bulk level, including the presence of a subclone of cells with evidence of whole-genome doubling, observed in patients 2 (10% of CTCs) and 4 (6%). These reproducible CNV patterns were not evident in bulk sequencing analysis and only detectable due to the resolution afforded by single-cell sequencing. Such subclonal copy number alterations were most pronounced in patient 4, where appreciable CNV gains or losses were only detectable at the single-cell level and not in the bulk tissue.

In patient 3, single tumor cells derived from a fresh frozen (FF) liver biopsy exhibited identical copy number profiles as CTCs and unsupervised hierarchical clustering of CTC and tumor copy number profiles demonstrated clustering of these cells together.

### CTC analysis reveals significant inter-and intrapatient heterogeneity

**Figure 4:**
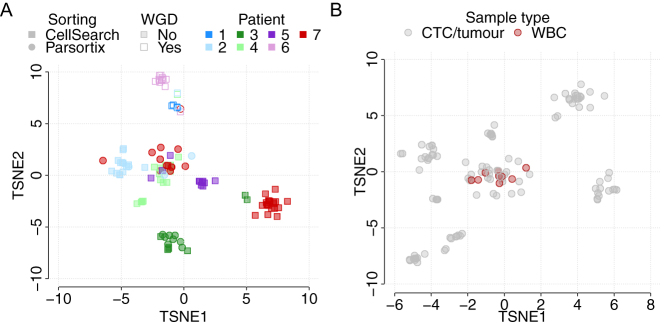
Relationship between CTCs from all seven NEN patients is revealed through TSNE analysis. (A) Single CTCs from all seven patients are visualized and can be identified by color in the phenobar at the top of the figure. Cells are also depicted according to enrichment strategy (see key). (B) TSNE of all analyzed CTCs and WBC.

To fully explore inter- and intrapatient CNV heterogeneity in NEN patients, the full set of 125 single CTCs from seven patient samples were further interrogated (Fig. 3). Copy number losses were seen more frequently than amplifications; however, whole-genome doubling was detected in all CTCs derived from two patients (patients 1 and 6). Despite the preponderance of losses, the CNV patterns of individual patients are dissimilar and this remains the case when considering only those patients of the small intestinal primary site (patients 1–4). These patient-specific patterns of CNV were confirmed using *t*-distributed stochastic neighbor embedding (TSNE; Fig. 4), which demonstrated clear clustering of individual patients, with no segregation according to the primary site. Conversely, all WBC clustered together regardless of patient of origin in keeping with their flat CNV profiles (Fig. 4B).

Within individual patients, there were observations of clonal CN alterations seen in 100% of CTCs, but also clear evidence of subclonal changes and individual cells with unique CNV profiles indicative of divergent evolution (Fig. 3). This intrapatient heterogeneity was only detectable at the single-cell level. The degree of intrapatient heterogeneity varied according to patient, with patients 3 and 6 demonstrating the highest average pairwise correlation of CTC CNV profiles, and hence the most homogenous copy number landscape across CTCs (Supplementary Fig. 2). However, the correlation of CNV profiles within patients remains higher than that observed between patients, underscoring the independent nature of CNV profiles originating in different patients, and the shared evolutionary history of CTCs, and thus CTC CNV profiles, within individual patients.

### CNV profiles vary according to enrichment strategy

**Figure 5:**
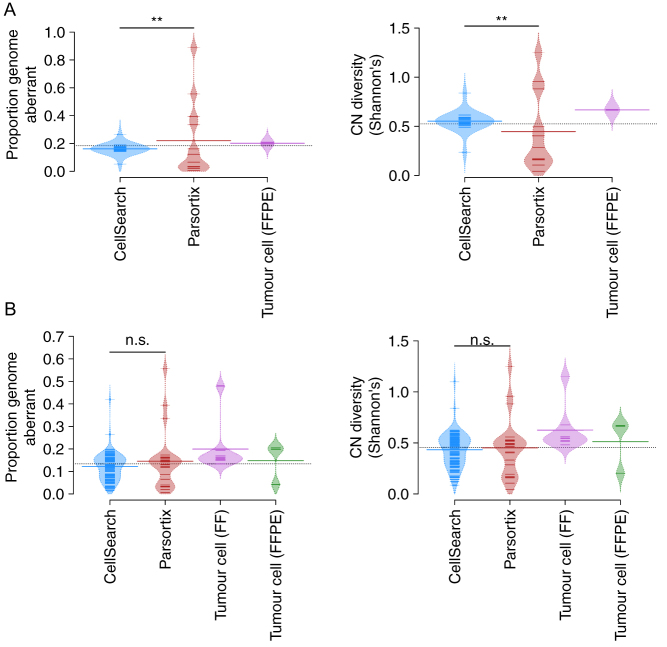
Distribution plot describing the impact of enrichment strategy in patient 7 (A) and all patients (B) on the proportion of the genome that is aberrant and CNV diversity as quantified by Shannon Index. Each small line represents the described value for a single CTC. Large bars represent mean values.

In patient 7, hierarchical clustering of CNV profiles demonstrated distinct clustering of CTCs enriched by the EpCAM-dependent CellSearch as compared to the epitope-independent, size-based Parsortix platform (Fig. 3). This is also demonstrated in Fig. 4 where Parsortix and CellSearch CTCs from patient 7 form largely separate groups. To investigate this further, we summarized single CTC profiles via two metrics; the proportion of the genome that is aberrant (copy number other than 2), and copy number diversity as enumerated by Shannon’s diversity index, and compared these metrics across cells according to the enrichment strategy utilized. There was a statistically significant difference in the distribution of both metrics between different enrichment strategies within patient 7 (Kolgomorov–Smirnov test, *P*  < 0.01, Fig. 5A), where Parsortix CTCs demonstrate a larger range in both metrics as compared to CellSearch CTCs, indicating greater cell-to-cell variation. Interestingly, the difference seen in patient 7 was not found to be statistically significant across all patients (Fig. 5B), indicating that these differences may vary on a patient-to-patient basis. This data suggests that restricting the analysis of CTCs to only those that express EpCAM may exclude subsets of tumor cells that could be clinically relevant.

### CTC molecular characterization

**Figure 6:**
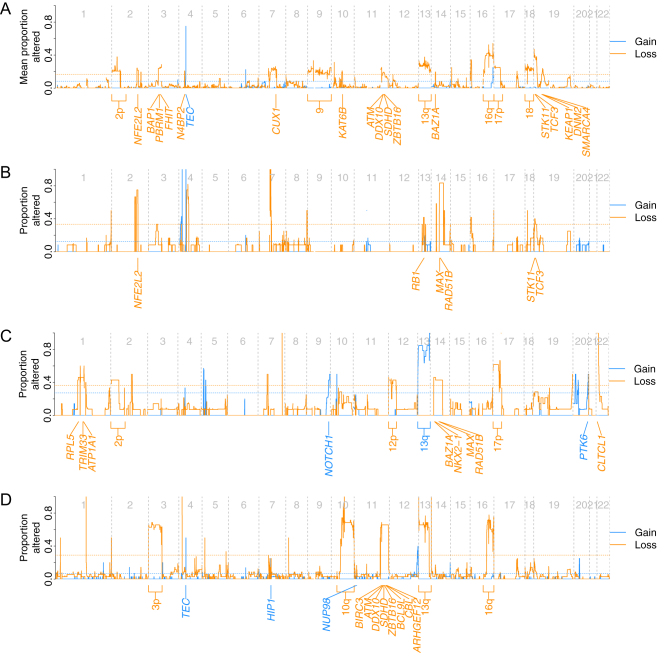
Frequency of genomic amplifications and deletions across all CTCs. Profiles demonstrated for SINET patients (A), patient 5; pancreatic NEN (B), patient 6; GOJ NEN (C), and patient 7; renal NEN (D).

In order to evaluate the clinical application of CTC CNV profiling as a surrogate for tissue biopsy, we interrogated CTC CNV profiles for prognostic or actionable copy number changes described in the NEN literature. Evaluation of the frequency of copy number amplifications and deletions within CTC CNV profiles from SINET patients revealed recurrent losses of chromosomes 9, 13q, 16q, and 18q (Fig. 6A). These have previously been described in SINETs supporting the technical reliability of our data and the potential use of CTCs as a tissue surrogate (Kulke *et al.* 2008, Banck *et al.* 2013, Hashemi *et al.* 2013, Karpathakis *et al.* 2016, Di Domenico *et al.* 2017). Of particular note is chromosome 18, loss of which is the most frequently reported genomic event in SINET, occurring in 60–78% of tumors and is of prognostic relevance (Karpathakis *et al.* 2016). Previously unreported alterations, including loss of chromosomes 2p and 7q22, were also identified. Although not reported in SINET, allelic losses in chromosome 2p are reported in colorectal, lung, and endometrial malignancies. The tumor suppressor gene *CUX1* is located at chromosome 7q22, knockdown of which causes increased PI3K signaling and AKT phosphorylation (Ramdzan & Nepveu 2014). This may be relevant in this patient population as deregulation of the PI3K/Akt/mTOR pathway is well-established in NEN, supported by the clinical efficacy of the mTOR inhibitor everolimus (Pavel *et al.* 2011, Yao *et al.* 2011).

Whole chromosome and arm gains at chromosome 4 have previously been described in SINET. We did not observe such large-scale gains, instead, we observed focal gains in the *TEC* gene on chromosome 4p12, which encodes a protein belonging to the Tec family of non-receptor protein-tyrosine kinases involved in the T-lymphocyte activation pathway and implicated in myelodysplastic syndrome.

CTCs from patient 7 (renal NET) demonstrated recurrent chromosomal alterations of likely clinical significance. Loss of chromosome 3p was observed in a high proportion of CTCs and harbors several tumor suppressor genes including the *VHL* gene at 3p25. Loss of heterozygosity (LOH) of 3p has been reported in the limited renal NET sequencing data available and is also found in over 90% of clear cell renal carcinoma (el-Naggar *et al.* 1995, Alimov *et al.* 2000). Loss of chromosomes 10q and 13q was also observed, the former of which encodes the tumor suppressor gene *PTEN* and is of prognostic relevance in renal cell carcinoma (Velickovic *et al.* 2002). Finally, as with SINET, chromosome 16q loss was frequently identified across patient 7 CTCs. Deletion of 16q is demonstrated across multiple malignancies, and LOH has been indicated as an early event in the development of breast and hepatocellular cancer with possible prognostic implications (Sakai *et al.* 1992, Hansen *et al.* 1998).

## Discussion

Copy number analysis of NEN CTCs confirmed a wide range of genomic aberrations making them readily distinguishable from WBC. All cells classified as WBC using the pre-determined DEPArray criteria demonstrated balanced copy number profiles, confirming the specificity and reproducibility of these criteria and accuracy of DEPArray sorting.

In this study, we show for the first time that somatic CNVs of NEN CTCs mirror those seen in FFPE tissue, validating these CTC enrichment and isolation technologies in NEN and confirming their potential use as a surrogate for tissue biopsy. The clinical applications of this finding have been demonstrated in other tumor types such as NSCLC, where good concordance between ALK-rearranged CTCs and ALK-positive tumor biopsies has been demonstrated (Pailler *et al.* 2013). This finding is particularly relevant in tumor types where tissue biopsy is not readily available or as in NEN, where the relatively good prognosis of patients with low-grade disease means surgical specimens or biopsies may have been taken several years previously and, therefore, not be representative of the current genomic landscape of the disease after multiple lines of systemic therapy. CTCs have the additional benefit of being non-invasive and, therefore, easily repeatable, thus allowing the monitoring of genomic change in real-time. Beyond this, serial CTC monitoring may also enable the detection of mechanisms of resistance (Pailler *et al.* 2015). Importantly, subclonal CNVs not discernible in bulk tissue analysis were detectable in single CTC samples thus allowing the identification of intrapatient genomic heterogeneity.

Unsupervised hierarchical clustering identified intrapatient genomic heterogeneity in NEN patients, with diverse single CTC CNV traces observed in some patients. The intrapatient CNV heterogeneity demonstrated in this study has also been observed in other tumor types such as prostate (Dago *et al.* 2014, Lambros *et al.* 2018) and colorectal (Heitzer *et al.* 2013) cancer. This is in contrast to lung adenocarcinoma, SCLC, breast, and gastric cancer, where more homogeneous CNVs have been observed in CTCs from individual patients (Ni *et al.* 2013, Heidary *et al.* 2014, Gao *et al.* 2017). Intrapatient heterogeneity is of clinical relevance as it may impact prognosis, response to treatment, and biomarker development. High intratumoral heterogeneity in tissue samples has been associated with a worse overall survival across different tumor types (Seol *et al.* 2012, Mroz *et al.* 2013). This relationship has not yet been examined with regards to the genomic profiling of CTCs, but low phenotypic diversity of prostate cancer CTCs has been shown to correlate with improved OS in patients treated with androgen receptor signaling inhibitors (ARSI), whereas high heterogeneity was associated with increased risk of death on ARSI relative to taxanes. Considerable heterogeneity was also demonstrated in CNV patterns between patients. This appears to be cancer-type specific. Ni *et al*. observed almost identical global CNV patterns in five different patients with lung adenocarcinoma with 78% of the gain and loss regions shared between any two patients (Ni *et al.* 2013), and similar findings have been reported in gastric cancer (Gao *et al.* 2017). However, increased inter-patient heterogeneity is seen in other tumor types, such as SCLC and breast cancer (Ni *et al.* 2013, Gao *et al.* 2017). The inter-patient heterogeneity in CNV profiles demonstrated in this study persists even when the analysis is confined to those patients with small intestinal primaries.

Epitope-dependent enrichment technologies such as the CellSearch platform limit recovery of CTCs to an EpCAM-positive subpopulation. In this study, we performed the first direct comparison of CTC CNV profiles using identical blood draws between the epitope-independent size-based Parsortix and EpCAM-based CellSearch. In patient 7, CTCs enriched using the CellSearch platform demonstrate reproducible CNV with high inter-cell concordance. However, CTCs enriched using Parsortix appear genomically distinct, lacking the conserved CNV demonstrated in CellSearch CTCs and displaying a wider range of inter-cell heterogeneity. Different methods of enrichment may, therefore, impact the results of single-cell genomic analysis and have implications for serial monitoring of CNV profiles. This finding is clinically significant as it may impact biomarker development. For example, a CNV-based classifier of CTCs has been shown to predict chemosensitivity in SCLC patients (Carter *et al.* 2017). In that study, all CTCs were enriched using CellSearch, and the classifier was less effective in those patients demonstrating intrapatient heterogeneity. The data presented in our study suggest that the efficacy of CNV-based classifiers such as this may be affected by the form of enrichment used and could not be directly extrapolated to CTCs enriched using alternative technologies. Furthermore, it suggests combining epitope-independent enrichment strategies with CellSearch may allow sampling of a wider population of CTCs with greater potential to fully capture CTC diversity.

SINET are characterized by a low mutational burden, with the most frequent mutation occurring in the cell cycle regulator CDKN1B (cyclin-dependent kinase inhibitor 1B) in only 8% of tumors (Francis *et al.* 2013, Crona *et al.* 2015). In this study, we identify recurrent loss of chromosome 18, the most common genomic event in SINET and predictive of PFS in SINET. Karpathakis *et al*. have previously demonstrated that CNV analysis of SINET primary tissue can be used to divide patients into three molecular subtypes with significant impact on PFS (Karpathakis *et al.* 2016). We also demonstrated novel and potentially targetable alterations such as focal gains in chromosome 4p12, which encodes the *TEC* gene (Yu & Smith 2011). Further work is required to validate this finding in a larger cohort of patients.

Despite the novel findings reported, we acknowledge some limitations; namely, the relatively low number of patients involved, as well as their heterogeneity in terms of grade and primary site. However, limiting the analysis to a smaller patient cohort allowed assessment of multiple CTCs per patient in order to better characterize intrapatient heterogeneity, whilst the overall large number of single cells analyzed allowed comparison with bulk tissue data and of cell enrichment techniques at the molecular level.

In conclusion, this is the first study to demonstrate that CNV analysis of single CTCs in NEN patients is feasible. We have demonstrated significant intra- and inter-patient genomic heterogeneity undetected by bulk tissue analysis. Additionally, we demonstrate for the first time, the presence of genomically distinct CTCs according to the enrichment strategy utilized, which has implications for the study of CTCs across all tumor types.

## Supplementary Material

Supplementary Figure 1. Cluster analysis of copy number profiles for CD45 positive cells reveals balanced copy number profiles. Each patient is depicted with one color as shown on the phenobar at the bottom of the heat map. Profiles are distinct from CTCs and in keeping with WBC populations.

Supplementary Figure 2. Average pairwise correlation for CNV profiles within (diagonal) and between (off-diagonal) patients. After adjusting for ploidy, there was low correlation between individual patients. The degree of heterogeneity varied on a per patient basis, with LPWGS demonstrating more homogenous CNV profiles and therefore lower intra-patient heterogeneity in patients 3 and 6.

## Declaration of interest

The authors declare that there is no conflict of interest that could be perceived as prejudicing the impartiality of the research reported.

## Funding

This work was supported by the CRUK & EPSRC Comprehensive Cancer Imaging Center, European Neuroendocrine Tumor Society Fellowship, University College London http://dx.doi.org/10.13039/501100000765 (UCL) CRUK, and NIHR Experimental Cancer Medicine Center Grant No. C12125/A15576, MRC award MR/M009033/1 and the UCL Hospitals NIHR Biomedical Research Center.
