# Supplementary figures and images for: Whole-genome sequencing of single circulating tumor cells from neuroendocrine neoplasms
Source: Endocr Relat Cancer. 2021 Jul 16;28(9):631–44. doi: 10.1530/ERC-21-0179 (PMC8428071; doi:10.1530/ERC-21-0179)

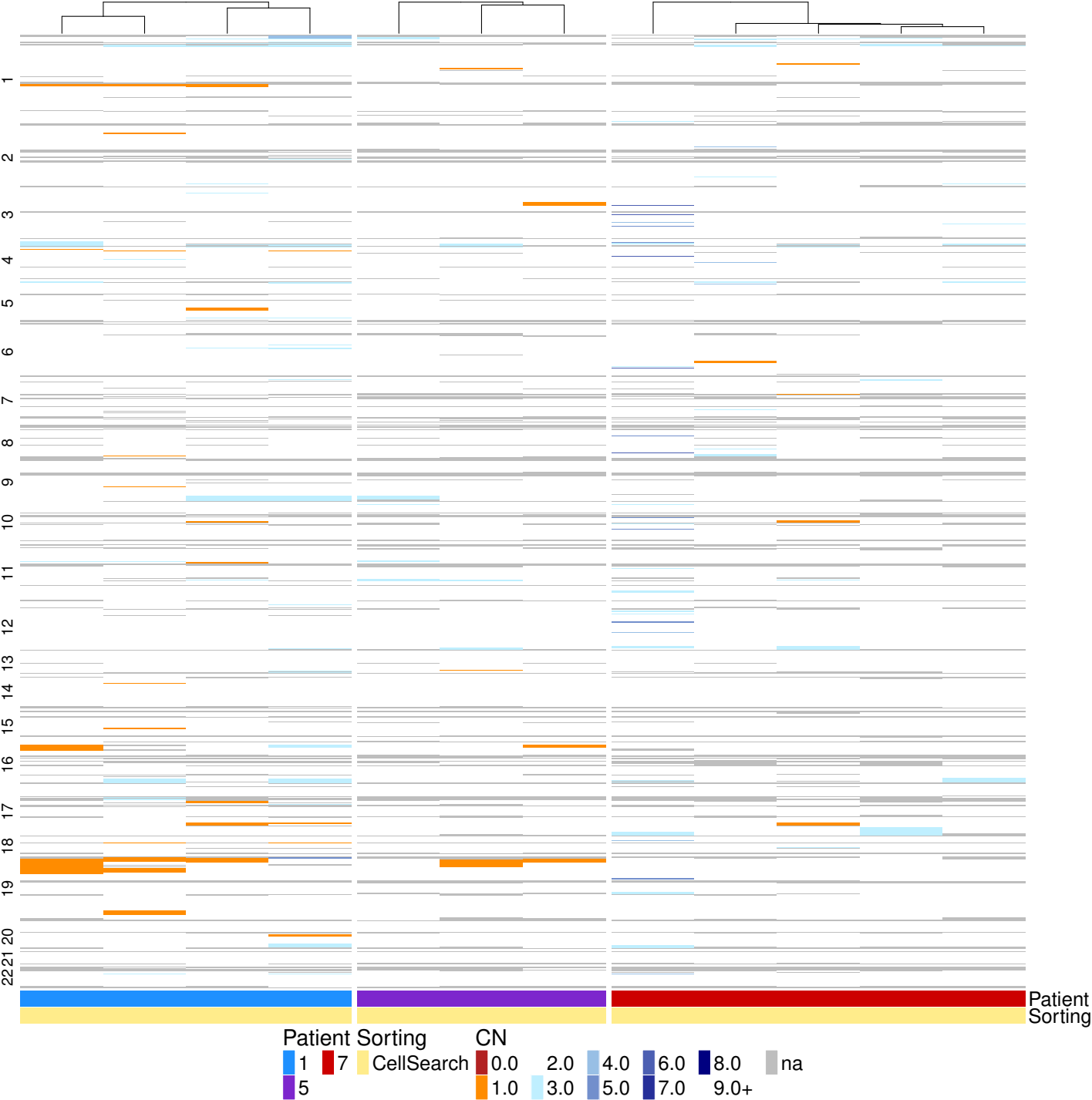

Supplement: Supplementary Figure 1. Cluster analysis of copy number profiles for CD45 positive cells reveals balanced copy number profiles. Each patient is depicted with one color as shown on the phenobar at the bottom of the heat map. Profiles are distinct from CTCs and in keeping with WBC populations. [file supplementary_figure_1.pdf]

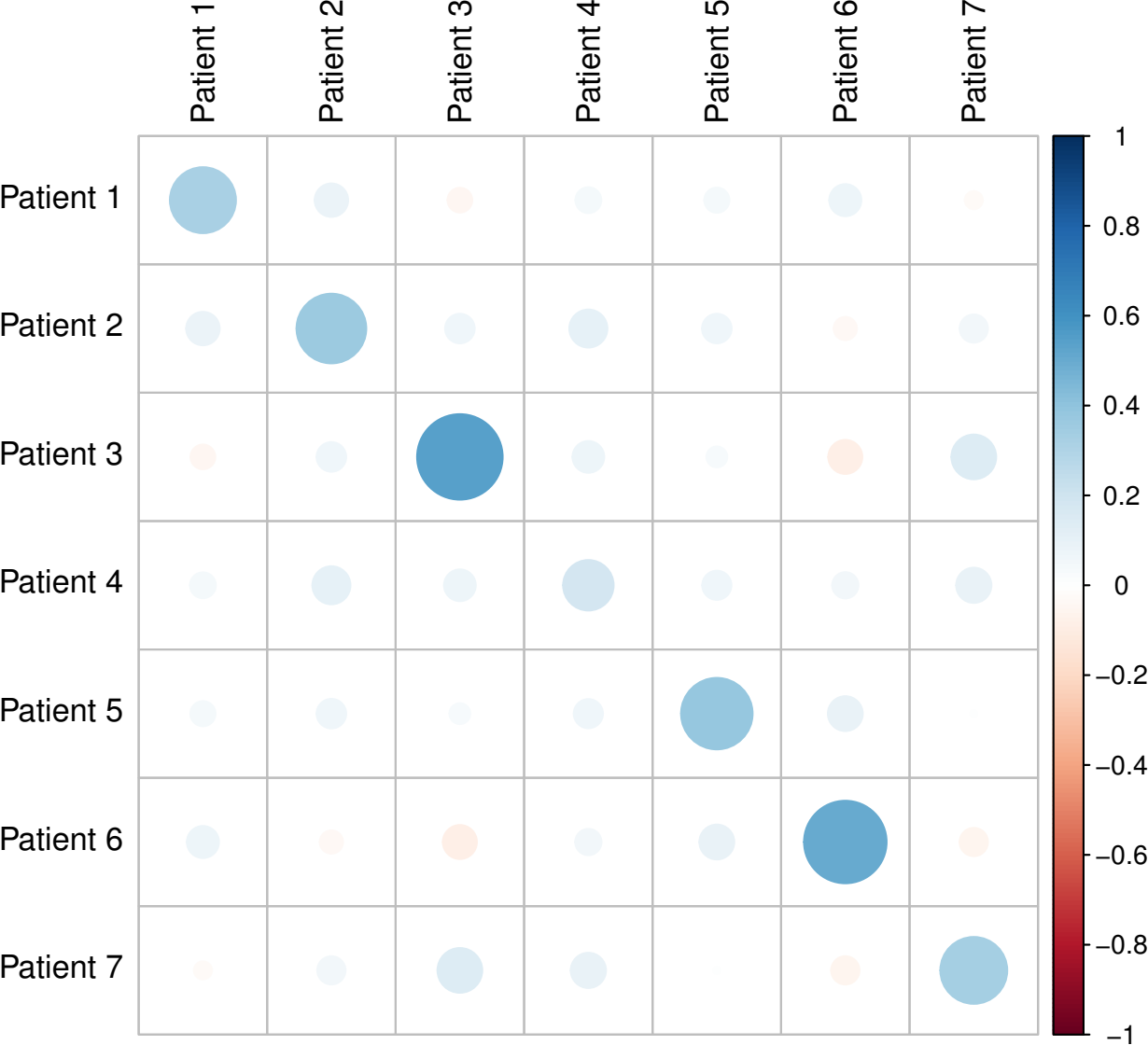

Supplement: Supplementary Figure 2. Average pairwise correlation for CNV profiles within (diagonal) and between (off-diagonal) patients. After adjusting for ploidy, there was low correlation between individual patients. The degree of heterogeneity varied on a per patient basis, with LPWGS demonstrating more hom [file supplementary_figure_2.pdf]
